# Assessing Service-Learning in Community-Based Veterinary Medicine as a Pedagogical Approach to Promoting Student Confidence in Addressing Access to Veterinary Care

**DOI:** 10.3389/fvets.2021.644556

**Published:** 2021-06-17

**Authors:** Erin King, Megan Mueller, Gregory Wolfus, Emily McCobb

**Affiliations:** ^1^Jonathan M. Tisch College of Civic Life, Tufts University, Medford, MA, United States; ^2^Department of Clinical Sciences, Center for Animals and Public Policy, Cummings School of Veterinary Medicine, Tufts University, North Grafton, MA, United States

**Keywords:** service-learning, accessible care, veterinary medicine, veterinary student, student confidence

## Abstract

Community-based veterinary medicine is a growing field, and veterinary students need to be able to work with clients facing complex barriers to receiving veterinary care for their pet. Many veterinary clients experience challenges accessing veterinary care due to financial limitations, transportation access, language comprehension, the ability to comply to the care plan (e.g., disabilities, physical, or mental health challenges, substance use), the ability to come to the clinic during the hours that it is open, and the ability to communicate outside of the appointment. The goal of this study was to assess student confidence levels working in accessible care before and after participating in a service-learning-based community veterinary rotation. Results show significantly higher student confidence levels for every barrier after completing the Tufts at Tech (TAT) Clinical Rotation at Cummings Veterinary School of Medicine. Additionally, 86% (*n* = 85) of students strongly agreed or agreed that TAT affected their thoughts about community medicine, and 77% (*n* = 76) strongly agreed or agreed that the rotation affected their feelings about underserved clients. Service-learning rotations in community-based veterinary medicine could be one pedagogical approach in training veterinary students to work with a diverse clientele.

## Introduction

Access to veterinary care in underserved communities is a long neglected topic that is finally beginning to gain wider attention within the veterinary and animal welfare fields. Community-based veterinary medicine describes veterinary clinic models that offer services and interventions for underserved or marginalized communities. Historically, accessible veterinary care has involved veterinary teaching hospitals, mobile, or low-cost spay/neuter clinics, and low-cost full-service clinics that focus on the financial strain of veterinary care (1). However, clients utilizing these services often face additional barriers to receiving care that likely coincide with disparities in human health care access, though very little research has focused on documenting these disparities (2). For example, low-income pet owners may rely on public transportation and therefore cannot travel to a veterinarian with their pet on the bus or subway due to pet restrictions (3). Other commonly identified barriers to veterinary care are veterinarian–client communication and relationships, cultural/language barriers, and lack of client education (1, 4). Preparing future veterinarians to work in community medicine and address a range of barriers to care facing clients is an important step in addressing issues of equity surrounding veterinary services.

In addition to efforts to make veterinary care more accessible to pet owners with economic constraints, the need for veterinarians to work and communicate effectively with clients from diverse communities is a pressing issue. Veterinary medicine continues to be one of the least diverse professions in the United States (5). According to the Bureau of Labor Statistics (6), in 2018, out of an estimated 102,000 veterinarians, 93% were white. In human medicine, minority health care professionals are more likely than non-minorities to serve medically underserved communities, thus increasing access to health care (7, 8). It is likely that racial disparities in veterinary medicine have lasting implications for diverse communities and perpetuate animal resource deserts in which very few or no services are provided to marginalized communities (3). While the North American Veterinary Medical Education Consortium (NAVMEC) Roadmap for Veterinary Medical Education in the 21st Century recommends that “veterinarians demonstrate an understanding of the manner in which culture and belief systems impact delivery of veterinary medical care while recognizing and appropriately addressing biases in themselves, in others, and in the process of veterinary medical care delivery” (9, p. 6), it is still unclear how best to teach cultural awareness in veterinary medicine. It is imperative that veterinarians understand how different belief systems may impact veterinary medicine and that we address implicit bias in healthcare services.

One pedagogical approach to training veterinary students to work with diverse communities and clients facing barriers to care is a service-learning experience through a community veterinary teaching model. Service-learning implies a meaningful, reflection-based, experiential learning process that involves both curricular and community involvement (10, 11). Service-learning aims to increase student awareness of stereotypes and assumptions while students begin to understand larger social issues that affect their service sites (12). It is important to stress the need for local community dictation of service needs and for programs to maintain an asset-based ideology that emphasizes strengths of communities and focuses on building relationships (13, 14). Service-learning strives to bring together the curricular components of education, hands-on applied learning skills, as well as long-term community relationships. Several studies suggest that participation in a service-learning program is associated with positive outcomes in five areas: attitudes toward self, civic engagement, social skills, attitudes toward school and learning, and academic achievement (15). For medical school students, those with increased exposure and experience practicing clinical skills and communication had higher confidence levels (16). More research is needed to understand how veterinary-specific service-learning programs may impact student outcomes. Increased exposure and experience communicating with clients in the veterinary community medicine field could lead to increased student confidence levels, which would support the use of service-learning programs in veterinary education.

At Cummings School of Veterinary Medicine at Tufts University, students participate in a 3-week service-learning clinical rotation at the Tufts at Tech (TAT) Community Veterinary Clinic. Through a partnership with the Worcester Technical High School in Massachusetts, TAT provides subsidized veterinary care to low-income pet owners in the Worcester, Massachusetts, area (17). This novel approach at TAT provides the opportunity for veterinary students to develop skills in primary care and general practice, including surgical skills, while enabling high school students to train as veterinary assistants. TAT works to provide veterinary students with experience practicing a spectrum of care principles while meeting community needs. Spectrum of care refers to the range of diagnostic and treatment options available for an animal that takes into consideration multiple factors such as cost, client, and veterinary education level, safety, and culture (18). One of the main goals of the TAT experience, beyond building clinical skills and working with a spectrum of care principles, is student reflection and thought about community medicine. This goal is accomplished through guided student reflection focus groups, as well as student review of performance with open communication. Often, in animal welfare, there is judgment held against low-income pet owners and implicit bias that negatively impact marginalized communities (3). Community medicine clinics are increasingly popular models to teach primary veterinary education, as well as a mechanism for analyzing internal bias in subsidized veterinary care models. Current literature has not explored the relationship between these models of teaching and student veterinary outcomes. Therefore, it is essential to explore how this pedagogical approach may support student attitudes and confidence levels.

The purpose of this study was to assess if participating in a service-learning, community-based veterinary medicine program fosters veterinary students' confidence in managing cases with clients facing barriers to veterinary care. A secondary aim of this study was to assess if this pedagogical approach shapes how students perceive community medicine and underserved veterinary clients. Service-learning models and pedagogical approaches for developing student confidence at managing clients with diverse needs have not been extensively studied in the context of veterinary medicine.

## Materials and Methods

### Procedures

Participants for this study were veterinary students in their third and fourth years of training entering a 3-week clinical service-learning rotation at the Tufts at Tech Community Veterinary Clinic in Worcester, Massachusetts. Students were sent a Qualtrics online survey *via* email 3 days before the start of their TAT clinical rotation and asked to complete another survey in person on the last day of their 3-week TAT rotation. Survey results were confidential and de-identified after pairing pre- and post-surveys. Data were collected from March 2019 to May 2020, and all study procedures were approved by the Tufts University Social-Behavioral-Educational Research Institutional Review Board (1903028). Students were not compensated for their participation in this voluntary research study, and participation did not affect a student's rotation performance evaluation.

### Measures

The pre-rotation survey asked basic demographic questions as well as their prior experience with community medicine programs. Examples of these community programs were public housing outreach, community cat clinics, and other non-Tufts community medicine clinics. Both surveys asked students if they had worked with clients experiencing any of the following barriers to receiving veterinary care: “financial limitations,” “transportation to the clinic,” “language comprehension,” “ability to comply with care plan due to client's disabilities (e.g., physical or mental health challenges, substance use),” “ability to come to the clinic during the hours that it is open (e.g., due to work schedule, family care responsibilities, need for assistance),” “ability to communicate with an owner outside of the appointment (e.g., to confirm appointments, check on medical progress, follow-up care),” and “other (please specify).” These measures were based on TAT client data and perceived barriers to care (19).

In the pre- and post-surveys, participants were also asked to rank their confidence levels from 1 to 5 (1 = not confident at all, 2 = slightly confident, 3 = somewhat confident, 4 = confident, 5 = very confident) about their ability to communicate with a client to manage barriers to care due to the following: “financial limitations,” “transportation to the clinic,” “language comprehension,” “ability to comply with care plan due to client's disabilities (e.g., physical or mental health challenges, substance use),” “ability to come to the clinic during the hours that it is open (e.g., due to work schedule, family care responsibilities, need for assistance),” “ability to communicate with an owner outside of the appointment (e.g., to confirm appointments, check on medical progress, follow-up care).” The post-survey also asked students to rank from 1 (strongly disagree) to 5 (strongly agree) how much they agreed/disagreed with the idea that the TAT rotation affected their thoughts about community medicine as well as feelings about underserved clients. Students were also asked what area of practice they were most likely to enter after graduation and could select the following options: small-animal internship, small-animal general practice, large-animal internship, large- or mixed-animal general practice, lab animal medicine, community or shelter medicine, zoo animal/wildlife medicine, or other.

### Analysis Plan

In order to understand if participating in a service-learning veterinary program increases student confidence, paired-samples *t*-tests were used to compare confidence levels between pre- and post-rotation for students who completed both surveys. Independent-samples *t*-tests were used to compare differences in confidence levels between students with and without experience with barriers to care before TAT. Student confidence was measured on a 5-point Likert scale for each barrier, which was treated as a continuous variable for the purpose of analysis. The distribution of each variable was normally distributed and analyzed for normality using histograms and central measures of tendency. All skewness and kurtosis levels were in the normal range between −1 and 1. All statistical analyses were performed with IBM SPSS 25.0 computer software.

## Results

Participants completed a pre-survey (*n* = 63) and post-survey (*n* = 99) before and after the clinical rotation, with 55 participants completing the survey at both time points. Demographic data were collected from participants in the pre-survey, and participants were 78% female (*n* = 49) and 22% male (*n* = 14). Students ranged from 24 to 40 years of age, with an average age of 26 years. Participants were 10% Hispanic or Latino/Latinx (*n* = 6) and 90% Non-Hispanic or Latino/Latinx (*n* = 56); 87% white (*n* = 54), 7% Asian (*n* = 4), 2% Black or African American (*n* = 1), and 5% other (*n* = 3). This sample is comparable to the entire 2020 Cummings graduating veterinary class that has a racial and ethnic composition of 78% (*n* = 72) white, 3% Hispanic or Latino/Latinx (*n* = 3), 5% (*n* = 5) Asian, 1% (*n* = 1) Black or African American, 1% (*n* = 1) two or more races, and 11% (*n* = 10) unknown.

In order to understand the range of student involvement in community medicine programs prior to TAT, all of the completed pre-surveys (*n* = 63) were analyzed for program experience. Here, 16% (*n* = 10) of the students had never participated in a community medicine-type program before, but 84% (*n* = 53) of the students had participated in at least one experience. Of these 53 students who completed the pre-rotation survey, 46% (*n* = 29) participated in one program prior to starting their TAT rotation, 21% (*n* = 13) participated in two different programs, 11% (*n* = 7) participated in three different programs, and 6% (*n* = 4) participated in at least four or more different community programs.

Questionnaires of participants who completed both the pre- and post-survey (*n* = 55) were analyzed to understand experience levels and confidence working with clients managing barriers to care. Prior to their TAT rotation, 89% (*n* = 49) of the students had experience working with clients experiencing financial limitations. The frequency of student experience with other barriers to care is shown in Table 1. After the TAT rotation, 100% (*n* = 55) of the students had experience working with clients with financial limitations. Additional exposure and experience with barriers after TAT can be found in Table 1.

**Table 1:** Percentage of students with experience managing barriers to care before and after participating in a service-learning community medicine rotation.

**Barrier to care**	**Student experience with barrier pre-rotation % (*n*)**	**Student experience with barrier post-rotation % (*n*)**	**Percent change**
Financial limitations	89.1% (49)	100% (55)	10.9%
Transportation	38.2% (21)	80% (44)	41.8%
Language	63.6% (35)	85.5% (47)	21.9%
Disability/condition	38.2% (21)	54.5% (30)	16.3%
Schedule	36.4% (20)	72.7% (40)	36.3%
Outside communication	36.4% (20)	87.3% (48)	50.9%

**Figure 1:**
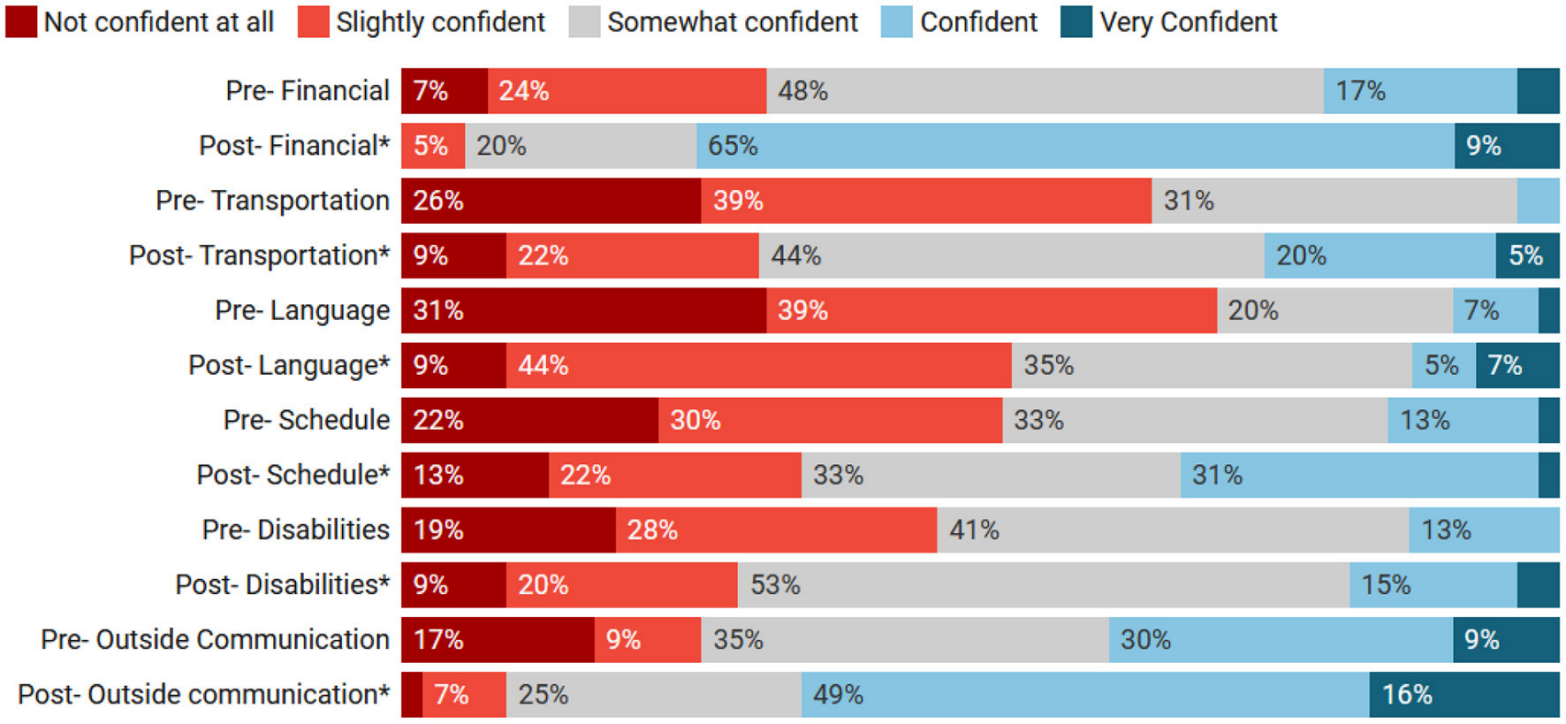
Percentages of student confidence pre and post tufts at tech (TAT). *Pre and post-confidence levels are statistically significant.

Pre and post levels of confidence are displayed in Figure 1. Paired-samples *t*-tests were performed to analyze student confidence pre and post the TAT rotation. Student confidence managing cases with barriers was significantly higher in each category after completing the TAT service-learning experience. The average level of confidence for managing cases with financial limitations prior to TAT was 2.9 (SD = 0.92), and after completing the rotation, confidence levels were significantly higher with an average of 3.8 (SD = 0.69), *t*_(53)_ = 8.29, *p* < 0.001. The *t*-tests for additional barriers can be found in Table 2.

**Table 2:** Student confidence in managing barriers to care before and after participating in a service-learning community medicine rotation.

		***N***	***M***	**SD**	**Standard error mean**	***t***	***df***	***p***
Financial	Pre-rotation	54	2.85	0.92	0.13	8.29	53	<0.001
	Post-rotation	54	3.78	0.69	0.09			
Transportation	Pre-rotation	54	2.13	0.85	0.12	5.81	53	<0.001
	Post-rotation	54	2.91	1.01	0.14			
Language	Pre-rotation	54	2.09	1.00	0.14	4.00	53	<0.001
	Post-rotation	54	2.57	1.00	0.14			
Disability/condition	Pre-rotation	54	2.48	0.95	0.13	2.65	53	0.01
	Post-rotation	54	2.83	0.93	0.13			
Schedule	Pre-rotation	54	2.43	1.03	0.14	2.70	53	<0.001
	Post-rotation	54	2.85	1.05	0.14			
Outside communication	Pre-rotation	54	3.06	1.20	0.16	4.62	53	<0.001
	Post-rotation	54	3.70	0.90	0.12			

Questionnaires for student confidence levels (*n* = 54, one participant left this section of the survey blank) were analyzed with independent-samples *t*-tests to compare the confidence levels of students who had exposure to barriers while at TAT and students who did not. The confidence level of students who had experience at TAT working with transportation barriers (*M* = 3.1, SD = 1.00) was significantly higher than that of students who did not have experience working with clients with transportation barriers during their time at TAT (*M* = 2.4, SD = 1.08), *t*_(97)_ = 2.85, *p* = 0.005. Similarly, confidence levels about barriers with regard to client disability were also significantly higher, *t*_(97)_ = 2.77, *p* = 0.007, when students had exposure to the barrier at TAT (*M* = 3.1, SD = 0.93) compared to those when students did not have exposure to clients with disabilities while at TAT (*M* = 2.6, SD = 0.76). Confidence levels for students with exposure to the other barriers at TAT were not significantly different [language: *t*_(97)_ = 1.41, *p* = 0.163, ability to come to the clinic during open hours: *t*_(97)_ = −1.15, *p* = 0.253, communication with owner outside of clinic *t*_(97)_ = −1.12, *p* = 0.264]. Experience working with barriers prior to TAT was related to initial student confidence in two areas before entering the TAT clinic. Students who had experience working with language barriers (*M* = 2.4, SD = 1.06) were significantly more confident than students who had no language barrier experience (*M* = 1.6, SD = 0.61), *F*_(57.11)_ = 7.28, *p* = 0.003. Additionally, students who had experience working with client disability (*M* = 2.9, SD = 0.85) were significantly more confident than students who had no disability barrier experience (*M* = 2.2, SD = 0.94), *t*_(52)_ = −2.43, *p* = 0.018.

All participants who completed the TAT rotation and took the post-survey (*n* = 99) were asked how strongly they agreed/disagreed with the following statement, “TAT affected my thoughts about community medicine,” 33% (*n* = 33) strongly agreed, 53% (*n* = 52) agreed, 11% (*n* = 11) neither agreed nor disagreed, 2% (*n* = 2) disagreed, and 1% (*n* = 1) strongly disagreed. Participants were also asked if they agreed or disagreed with the statement, “TAT affected my feelings about underserved clients,” in which 27% (*n* = 27) strongly agreed, 50% (*n* = 49) agreed, 21% (*n* = 21) neither agreed nor disagreed, 1% (*n* = 1) disagreed, and 1% (*n* = 1) strongly disagreed.

When looking at planned future career choices post the TAT experience (*n* = 89, 18 missing data points), 72% (*n* = 64) of students were interested in small-animal general practice or internships, with four of those students also being interested in community or shelter medicine. Here, 11% (*n* = 10) of students were interested in large-animal general practice/internship, 7% (*n* = 6) lab animal practice, 5% (*n* = 4) small-animal exotics, 1% wildlife/zoo medicine, and 4% (*n* = 4) other.

## Discussion

### Student Experience With Barriers to Care

Prior to entering the TAT rotation, many students had already participated in a number of community medicine programs and had some experience working with clients facing barriers to care. Due to the high cost of veterinary care, it is not surprising that 89% of students had prior experience working with clients facing financial limitations, particularly if student respondents generalize this question to refer to all clients struggling with the cost of care rather than restricting their exposure to clients in an underserved community. In addition, Cummings School of Veterinary Medicine includes content about working with underserved clients in the preclinical curriculum, and the Community Medicine Program offers a number of opportunities for preclinical students to get involved in community-based initiatives. These opportunities tend to be popular among students, as they provide a chance for students to get hands-on exposure to animals. Future research should investigate how veterinary students define and evaluate a client's financial limitations, and how appropriate responses to these limitations can be effectively addressed in veterinary school curriculum.

Financial cost of veterinary care is the most commonly cited barrier clients face at TAT (19), so it is understandable that 100% of students reported working with clients experiencing financial constraints. It is important to note that even though TAT works to provide subsidized services to clients through a low-cost model of care (prices are set at ~25% of the national average), financial constraints are still a predominant barrier to receiving veterinary care even at this clinic. The average cost transaction at TAT is $75, which is beyond the reach of many clients struggling to meet basic needs for housing, food, and transportation. Community medicine programs must continually work to address financial needs of the community members they serve, and at the same time, veterinary students need to develop a spectrum of care knowledge and confidence to work with clients facing financial limitations.

Beyond financial limitations, students managed cases with a range of barriers to care. Working with clients who spoke a different language was the second most common experience reported by students (88%), highlighting the need for veterinary medicine to have formal interpretation and translation services available for clients. Landau (20) found that only about 8% of small-animal practice staff could communicate with Spanish-speaking pet owners. In addition to language services, veterinarians need to continuously work to develop cultural humility in order to address client needs and improve communication. The ability for students to communicate with owners outside of the clinic (get a hold of someone *via* phone or email) was also a considerable barrier, with 87% of students experiencing this issue in their case management as shown in Table 1. Clients with a low income who utilize TAT may potentially have more difficulty accessing consistent lines of communication due to limited access to Internet and/or cell phone services. The ability to communicate with owners about the treatment plan, follow up care, and schedule future appointments is vital to sustainable veterinary—client relationships and an important factor to consider in community medicine. By participating in TAT, students are encouraged to examine these deeper issues of service accessibility and external factors that may impact clients and patients.

### Student Confidence

Students reported significantly higher confidence in managing cases with barriers to care after their service-learning experience. Overall, students were most confident about managing financial limitations. This finding was expected because students encountered this limitation most often and were able to develop a stronger skill set and become more comfortable working within the spectrum of care. In order to help address the financial burden of clients, TAT offers a Patient Care Fund that students can utilize to assist clients who are facing financial hardship. Students are given the option to use the funds for their own patients or those of their peers. This fund could be a factor in student confidence, and more research is needed on how these funds may contribute to student decision-making.

Conversely, students were least confident managing cases with a language barrier, which again highlights the need for more formal interpretation and translation services in veterinary medicine, as well as the importance of training for students to work with interpreters. Moreover, interpretation was one area where TAT lacks a formalized program to provide client assistance, meaning students had to rely on *ad hoc* strategies using bilingual team members to assist non-English-speaking clients.

While confidence in all areas improved post TAT, students who had specific experiences managing cases with transportation barriers and clients with disabilities while at TAT were significantly more confident in those two areas than students who did not have those experiences. It is possible that the fact that TAT has a cab voucher program to support clients who need assistance with transportation helped students become more confident in dealing with clients with transportation as a barrier because there was a specific resource to offer. In addition, because TAT has a program to address this need in the community, students are now able to see more clients with transportation challenges who otherwise would not have been to the clinic, thus boosting their confidence through exposure.

Other barriers that students were exposed to at TAT included language difference and clients with disabilities. Students who had experience with these particular barriers prior to the TAT rotation had higher confidence levels with those barriers than students without the experience at the start of the rotation. However, this prior experience working with barriers did not impact student confidence levels post rotation. We conclude that while prior experience with barriers to care may influence initial student confidence, this service-learning experience impacts students in a way that increases student confidence after the rotation regardless of prior experience in other community medicine programs. This finding has important implications for other schools or organizations wishing to start similar programs, in that while the program at TAT is part of a curriculum-wide effort to increase student familiarity with care barriers, it is apparent that it is the exposure to the clients themselves that is the most impactful experience for the students.

### Student Attitudes

After participating in TAT service-learning, 86% (*n* = 85) of students strongly agreed or agreed that TAT affected their thoughts about community medicine and 77% (*n* = 76) strongly agreed or agreed that the rotation affected their feelings about underserved clients. This rotation enables students to interact with community members in a way that encourages them to critically examine their own held biases and attitudes toward underserved communities. While most students were not planning to enter a career in community medicine (either before or after their rotation), it is important for all future veterinarians to have a broader understanding of diverse communities and client needs. Future research should address how and in what way student attitudes and feelings were affected by TAT, and if those changes are sustained throughout their career. Each student may have been impacted by the rotation in different ways; therefore, it is important to explore this topic further. Additionally, the guided reflection focus groups that TAT provides for students may impact student learning and attitudes toward community medicine. It could be that hearing peer reflections and having time to debrief about working in the community could be an important part of this pedagogical approach. Future studies should examine how reflection may impact learning in the veterinary setting and the mechanisms by which service-learning may increase confidence.

While survey results demonstrated the relationship between a service-learning rotation and student exposure to care barriers as well as student confidence in their ability to overcome such barriers for the client, this study has several limitations. First, the sample size of students who completed both the pre- and post-rotation assessment for this voluntary study was only about half of the total number of clinical year students. However, as this is a core rotation taken by all students, the demographics of our sample closely resemble those of the broader student population. In addition, this survey included only 1 year of veterinary students and was not formally piloted. Longitudinal data about how long confidence levels last or flux over time is not yet available. We also only sampled students at one service-learning site; future studies should compare different community clinic models and the impact on student learning in order to examine specific mechanisms of community-based learning. In addition, while student confidence was improved by this rotation, future studies should explore how and in what way students develop confidence and the ability for internal reflection. Future work would also benefit from the development of validated scales of student confidence in veterinary medicine settings. Finally, we relied only on the self-perception of confidence by students, and future research should include the perceptions of clients and professors as well as to develop a measure of competence for addressing barriers to care.

The ability of future veterinarians to understand systemic issues surrounding poverty that disproportionally impact underserved communities is critical to the implementation of successful treatment plans and long-term positive client relationships. A service-learning community medicine rotation, like TAT, may be an impactful way to teach students about the barriers to care that clients face while developing a stronger understanding of the spectrum of care principles. In addition, a surprising finding was that the service-learning rotation designed to expose students to clients with financial limitations was also an important clinical environment to train students to work with a wide variety of clients, including those with disabilities as well as limited English proficient clients. If the veterinary medical community is truly going to be accessible to all pets and their owners, it is essential that our industry and our educational systems develop resources to ensure that all can be served.

## Data Availability Statement

The datasets presented in this article are not readily available because data sharing must be approved by the Institutional Review Board. Requests to access the dataset should be directed to erin.king@tufts.edu.

## Ethics Statement

The studies involving human participants were reviewed and approved by Tufts University Social-Behavioral-Educational Research Institutional Review Board. The patients/participants provided their written informed consent to participate in this study.

## Author Contributions

The project was conceived by MM, EM, and GW. EM prepared the funding proposal. MM, GW, EM, and EK designed the survey instruments and secured IRB approval. EK administered the survey and collated the results. Initial data analysis was performed by EK with input from MM and EM. The manuscript was prepared by EK and reviewed by all authors with significant editorial assistance from MM and EM.

## Conflict of Interest

The authors declare that the research was conducted in the absence of any commercial or financial relationships that could be construed as a potential conflict of interest.
